# Supplementary material for: Distinct metabolic features of genetic liability to type 2 diabetes and coronary artery disease: a reverse Mendelian randomization study
Source: eBioMedicine. 2023 Mar 2;90:104503. doi: 10.1016/j.ebiom.2023.104503 (PMC10009453; doi:10.1016/j.ebiom.2023.104503)
Supplement: Supplementary Figures [file mmc3.pdf]

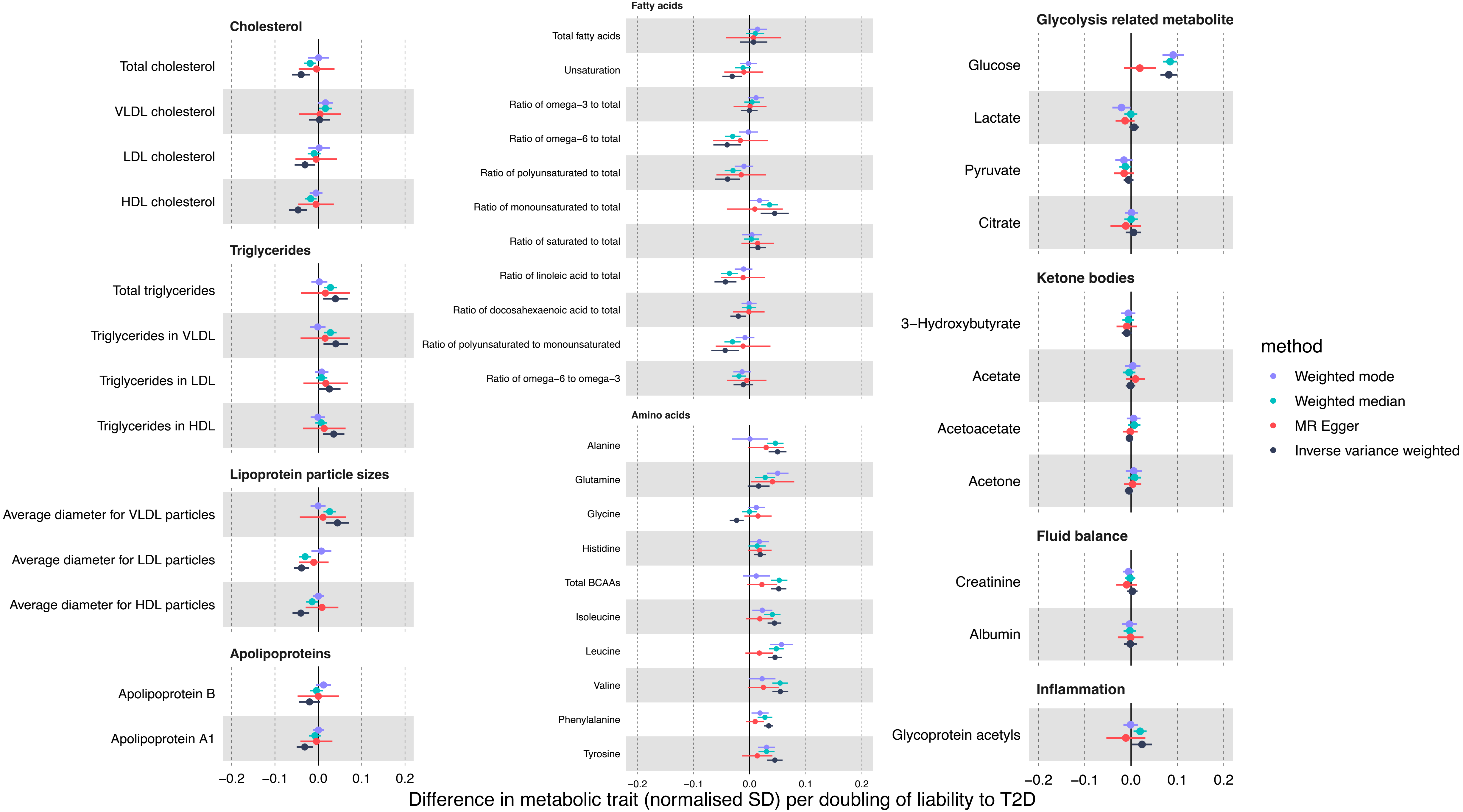

Supplementary Figure 1: Effect of T2D liability on metabolic traits. Effect estimates are normalised SD unit differences in metabolite per doubling of liability to T2D based on IVW, MR Egger, weighted median and weighted mode MR models.

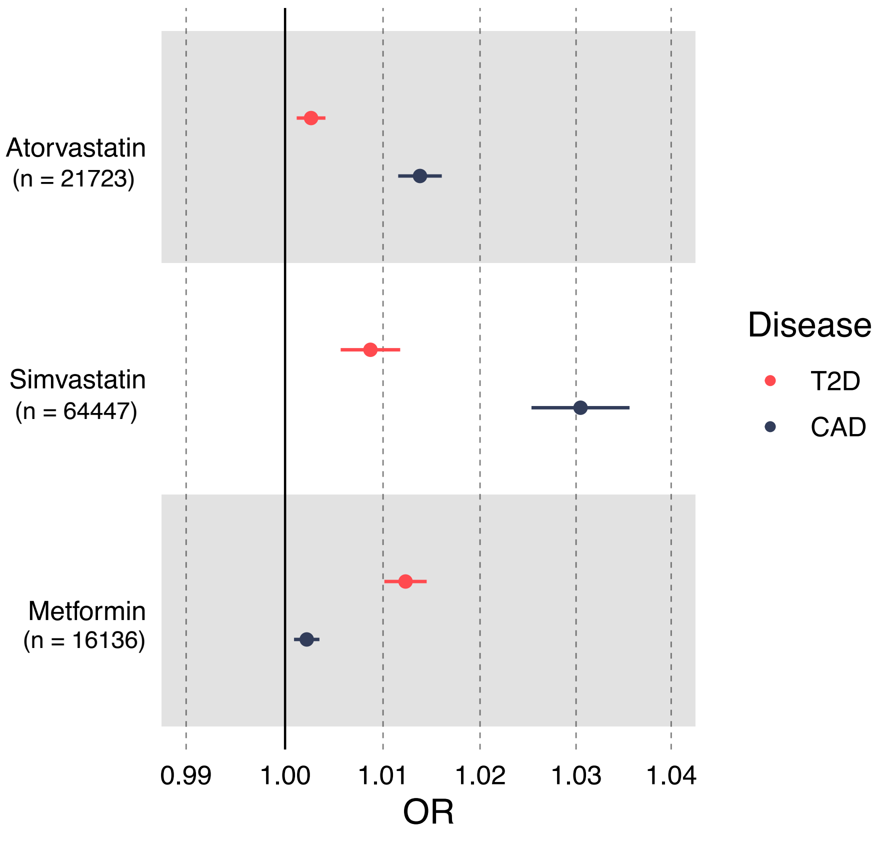

Supplementary Figure 2. Effect of T2D and CAD liability on statin and metformin use. Effect estimates are odds ratios when liability to disease is doubled, based on IVW models. T2D, type 2 diabetes; CAD, coronary artery disease; IVW, inverse variance weighted.

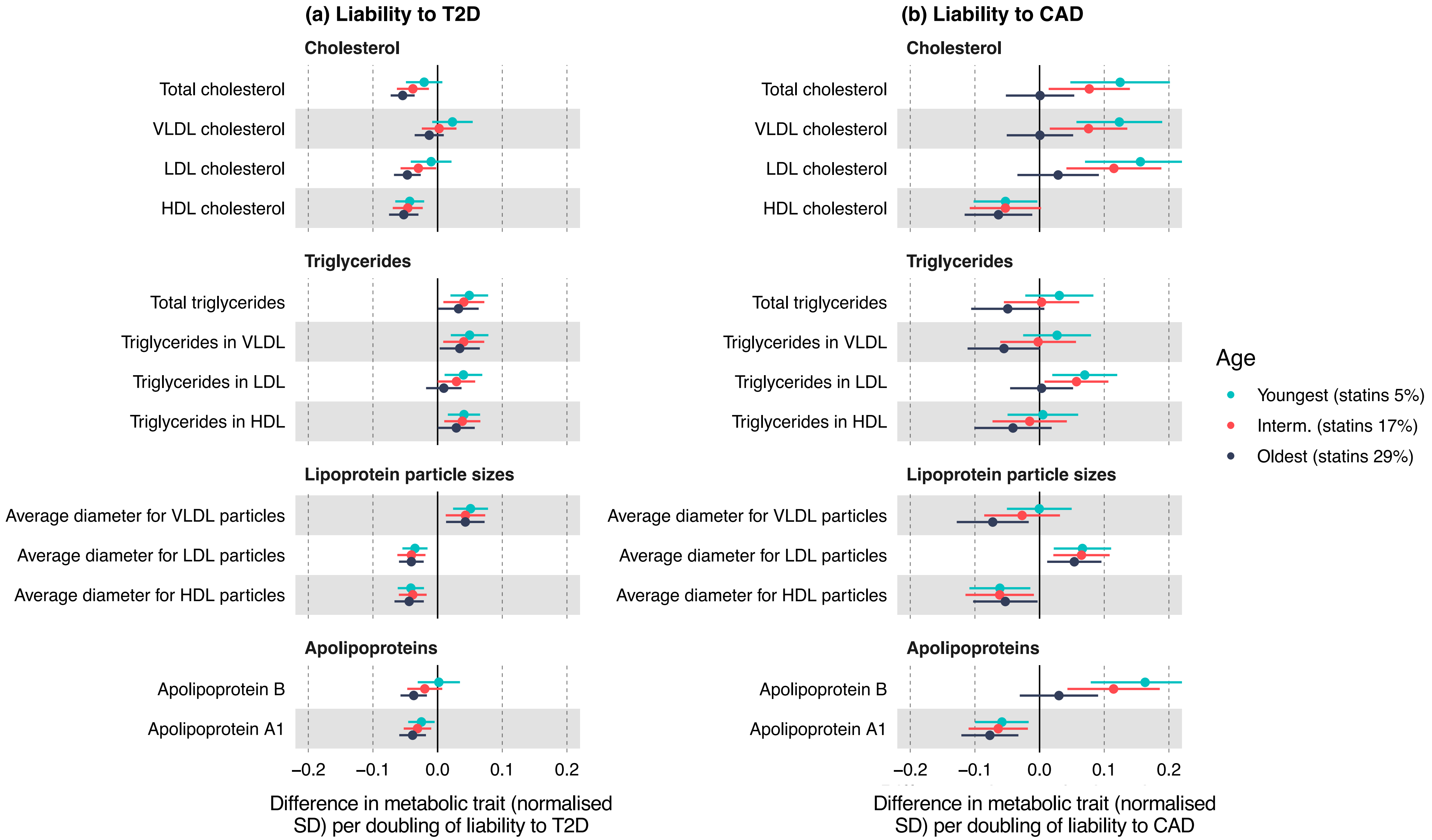

Supplementary Figure 3. Effect of **(a)** T2D and **(b)** CAD liability on lipids and lipoproteins in age tertiles. Effect estimates are normalised SD unit differences in metabolite per doubling of liability to disease based on IVW models. T2D, type 2 diabetes; CAD, coronary artery disease; SD, standard deviation; IVW, inverse variance weighted.

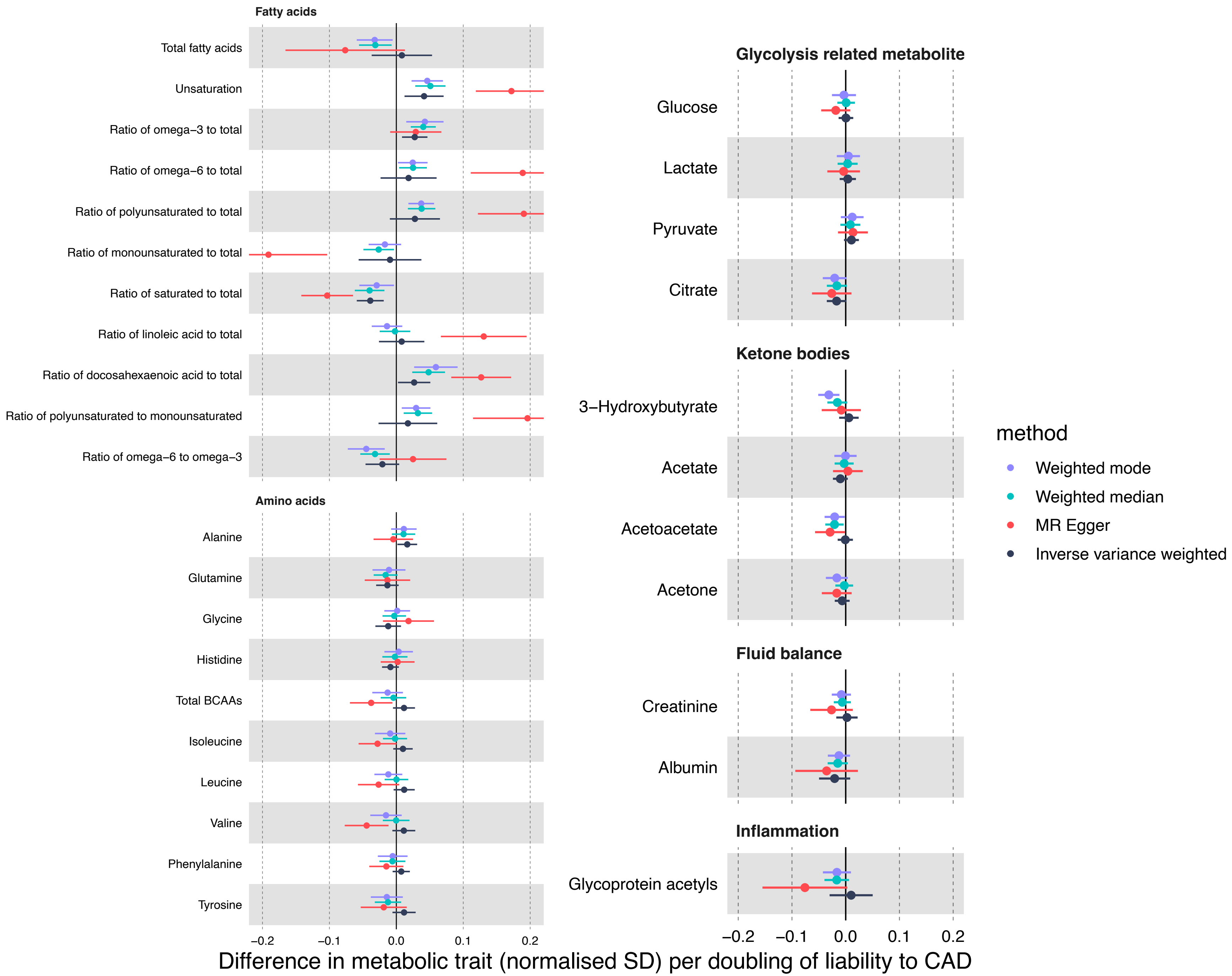

Supplementary Figure 4: Effect of CAD liability on metabolic traits. Effect estimates are normalised SD unit differences in metabolite per doubling of liability to CAD based on IVW, MR Egger, weighted median and weighted mode MR models.

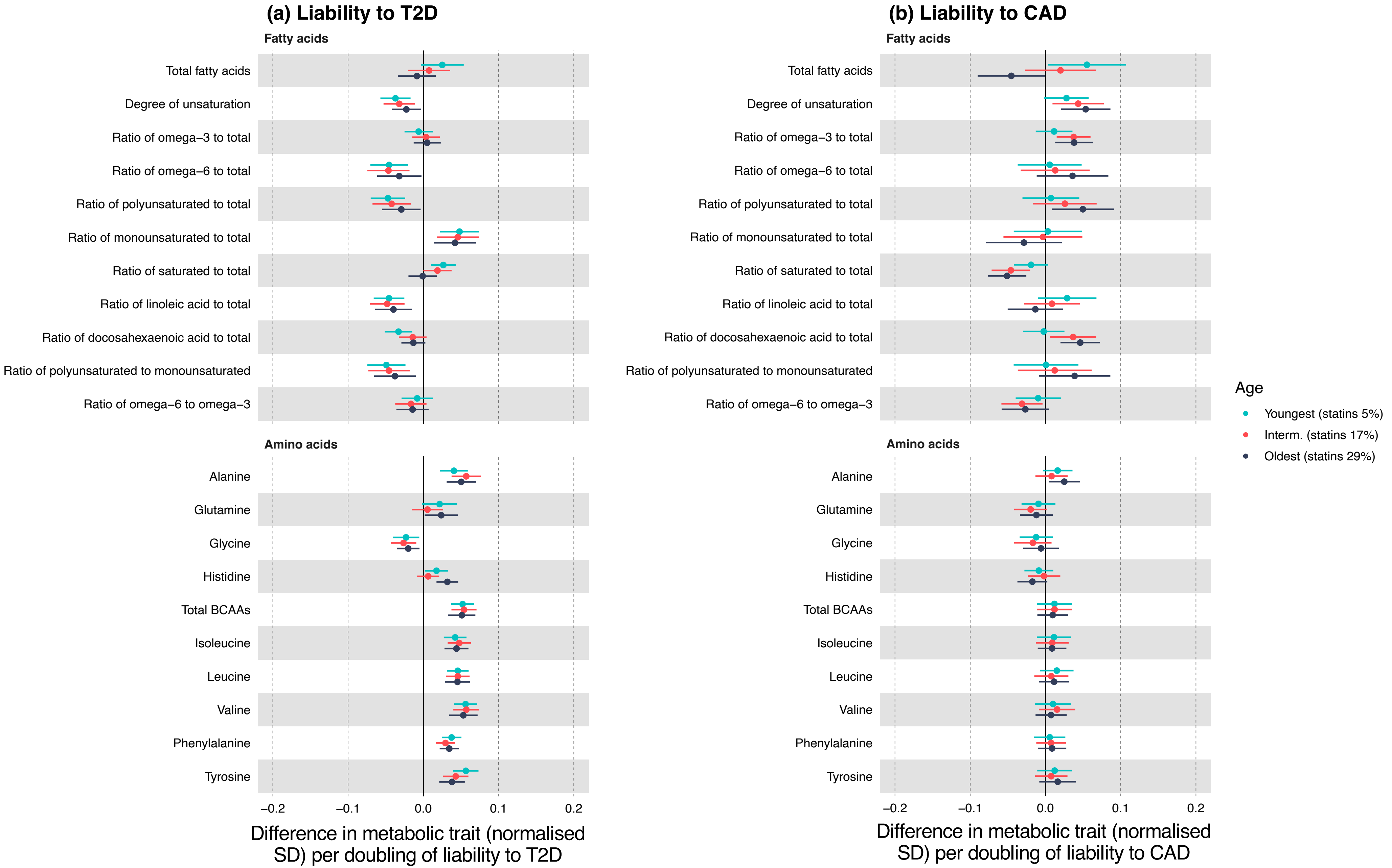

Supplementary Figure 5. Effect of **(a)** T2D and **(b)** CAD liability on fatty acids and amino acids in age tertiles. Effect estimates are normalised SD unit differences in metabolite per doubling of liability to disease based on IVW models. T2D, type 2 diabetes; CAD, coronary artery disease; SD, standard deviation; IVW, inverse variance weighted.

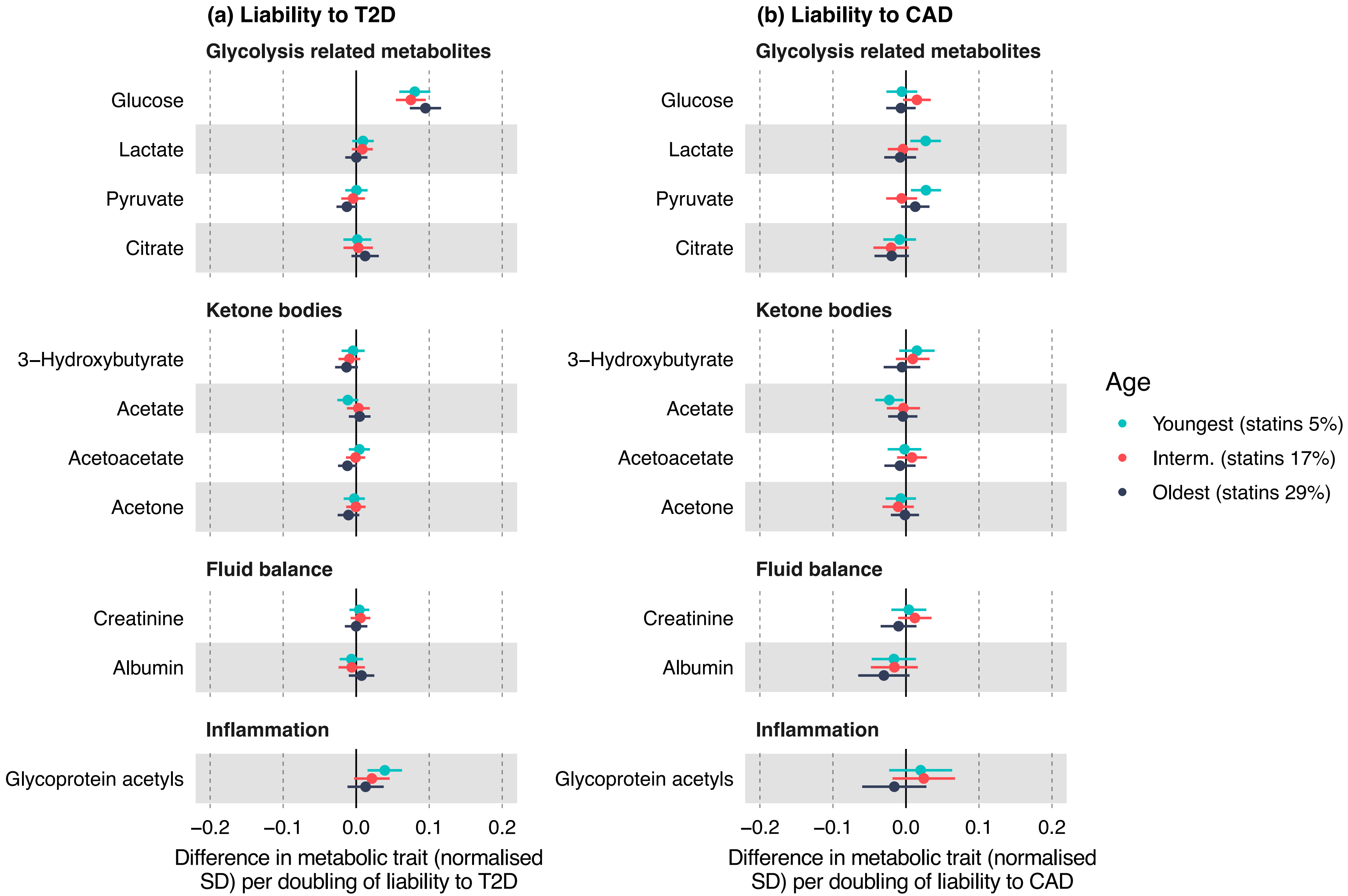

Supplementary Figure 6. Effect of **(a)** T2D and **(b)** CAD liability on glycolysis related metabolites, ketone bodies, fluid balance metabolites and glycoprotein acetyls in age tertiles. Effect estimates are normalised SD unit differences in metabolite per doubling of liability to disease based on IVW models. T2D, type 2 diabetes; CAD, coronary artery disease; SD, standard deviation; IVW, inverse variance weighted.

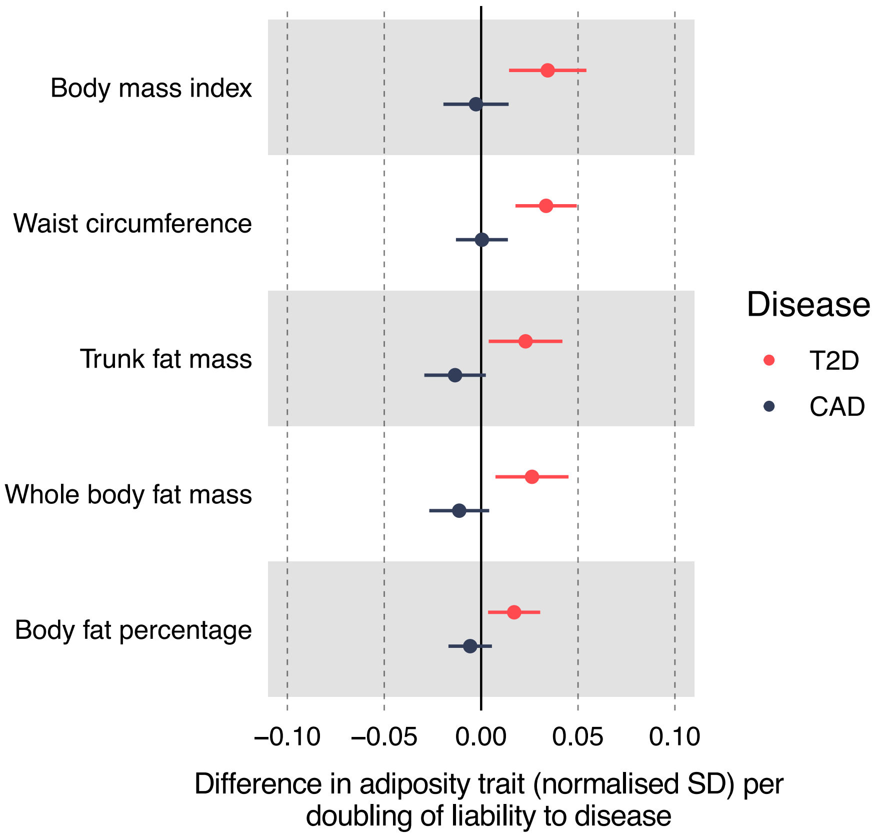

Supplementary Figure 7. Effect of T2D and CAD liability on adiposity. Effect estimates are normalised SD unit differences in adiposity trait per doubling of liability to T2D or CAD, based on IVW models. T2D, type 2 diabetes; CAD, coronary artery disease; SD, standard deviation; IVW, inverse variance weighted.

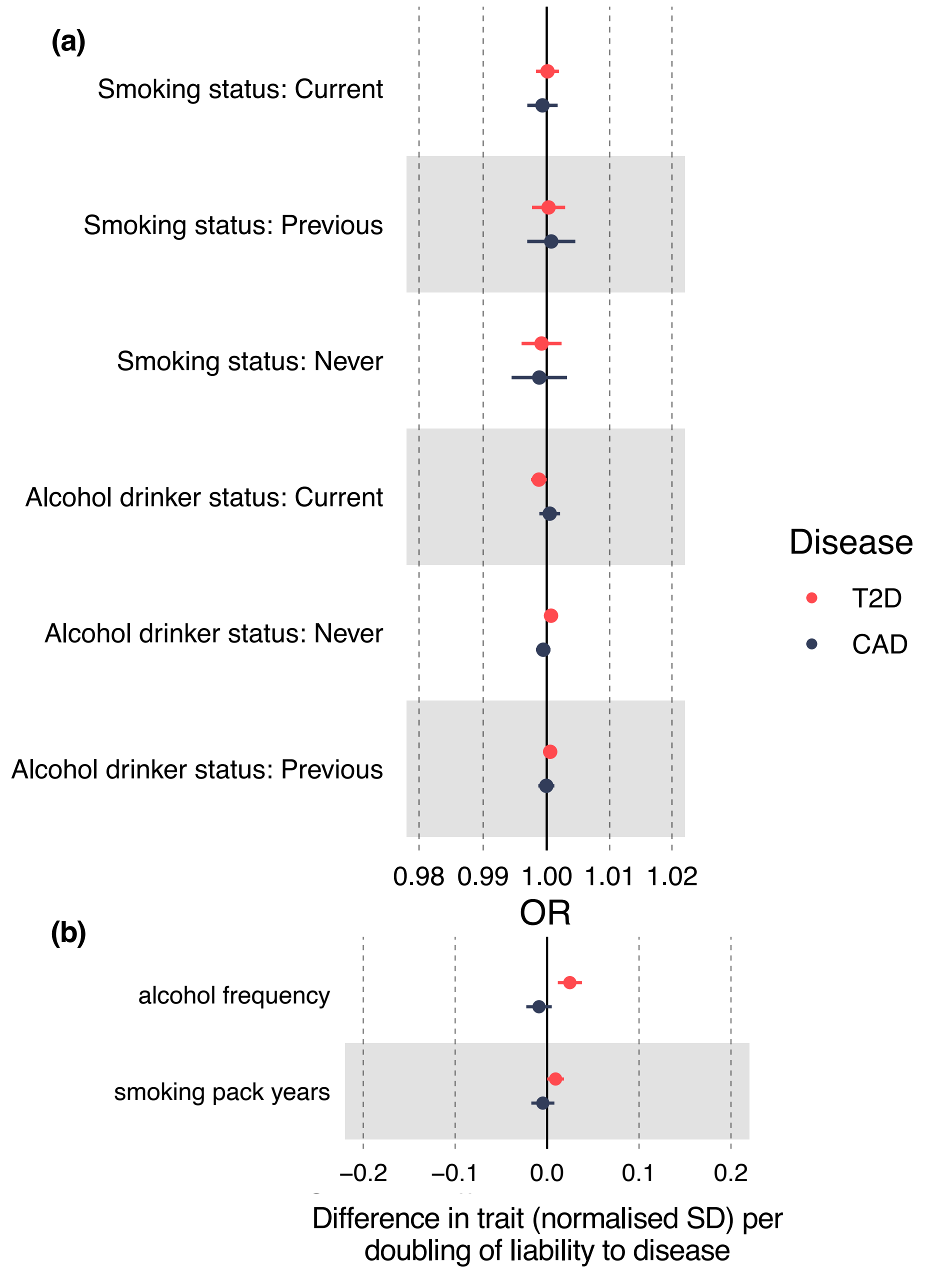

Supplementary Figure 8. Effect of T2D and CAD on smoking and alcohol behaviours. Effect estimates are odds ratios when disease liability is doubled in **(a)** and SD-unit differences in metabolite per doubling of liability to T2D or CAD in **(b)**, based on IVW models. T2D, type 2 diabetes; CAD, coronary artery disease; SD, standard deviation; IVW inverse variance weighted.

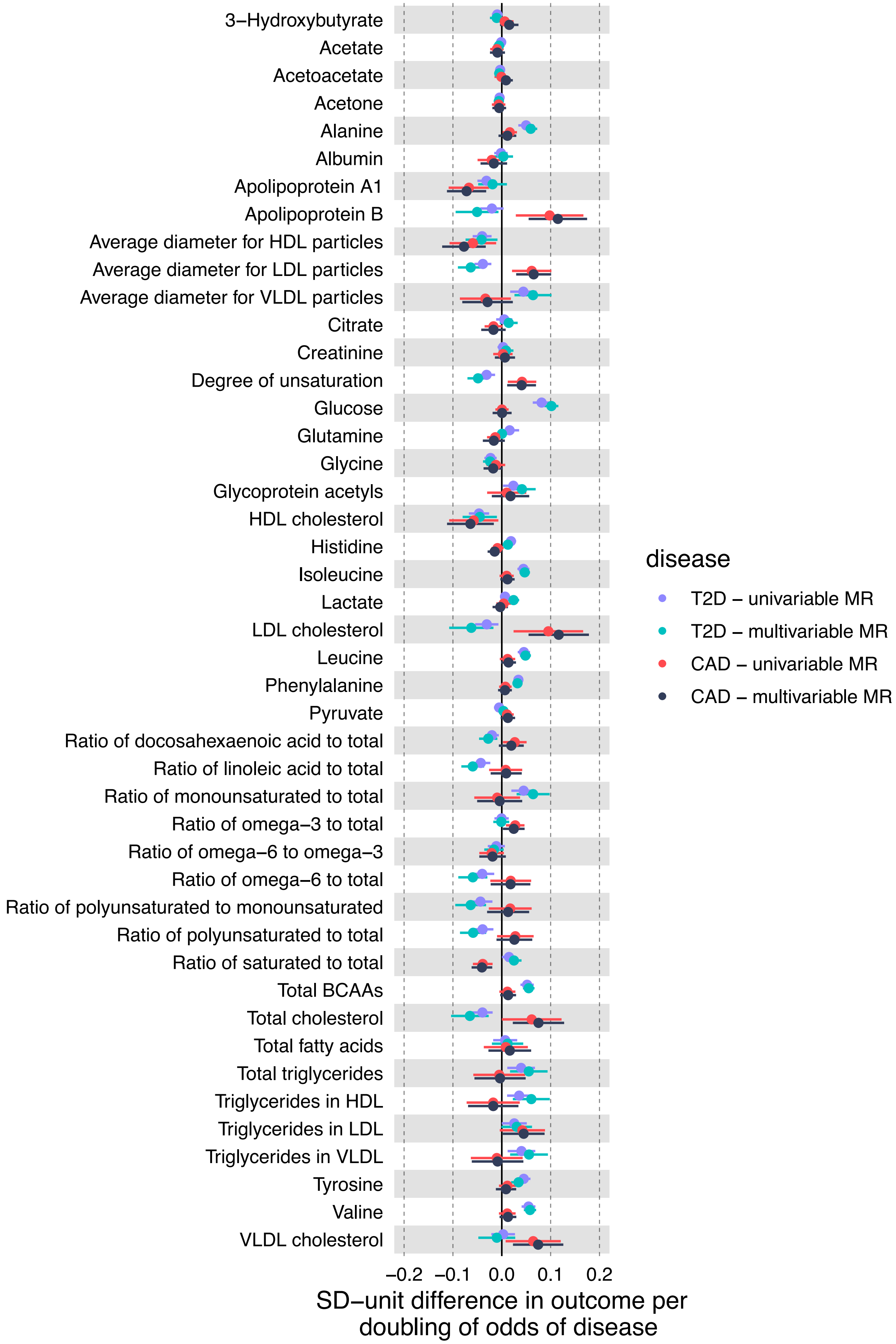

Supplementary Figure 9. Effect of T2D and CAD on metabolites. Effect estimates are SD-unit differences in metabolite per doubling of liability to T2D or CAD based on univariable and multivariable IVW models. Metabolites are in alphabetical order. T2D, type 2 diabetes; SD, standard deviation; IVW inverse variance weighted.

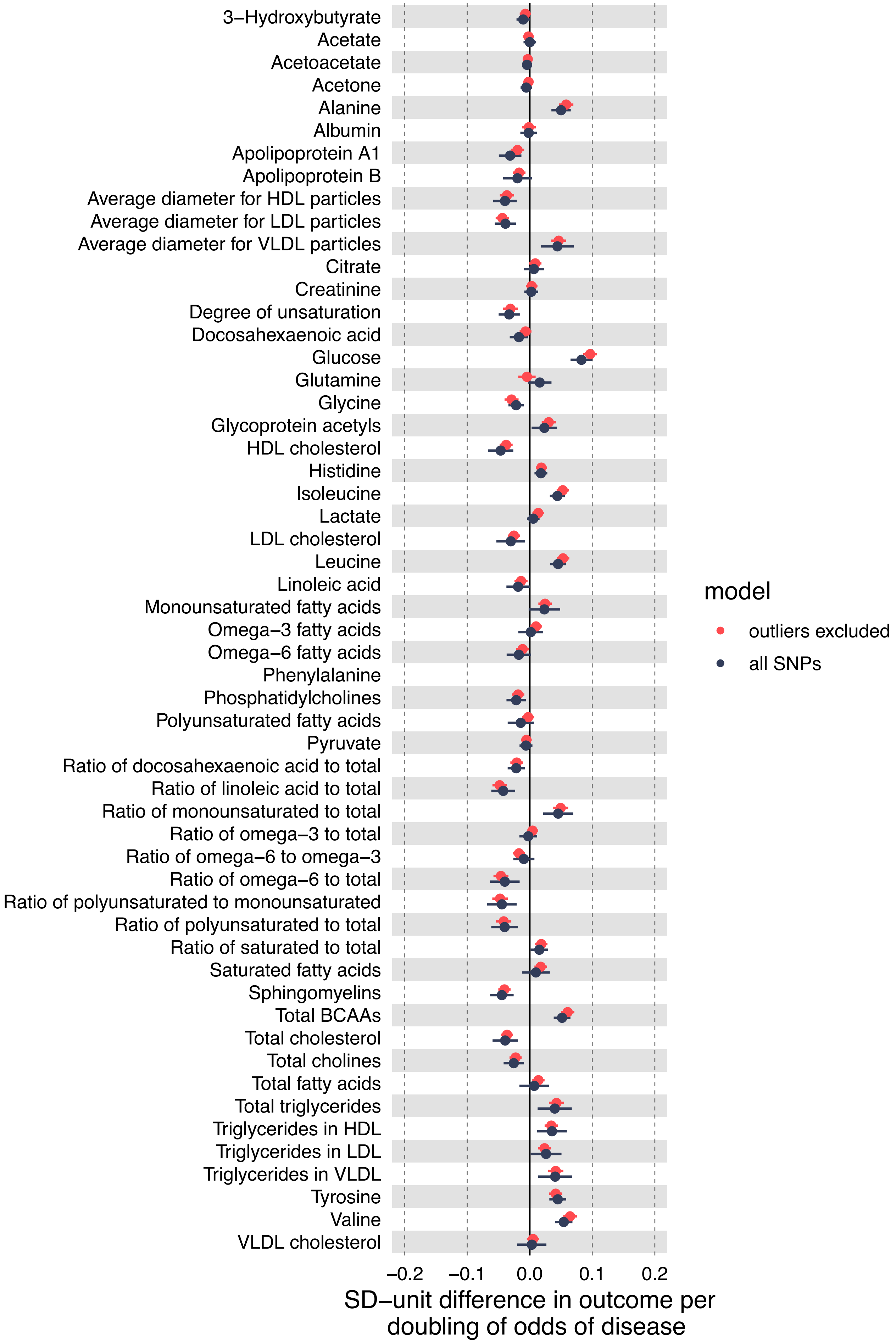

Supplementary Figure 10. Radial MR results of the effect of T2D on metabolites. Effect estimates are SD-unit differences in metabolite per doubling of liability to T2D based on IVW models, with all SNPs (blue) versus with outlying SNPs excluded (red). Metabolites are in alphabetical order. T2D, type 2 diabetes; SD, standard deviation; IVW inverse variance weighted.

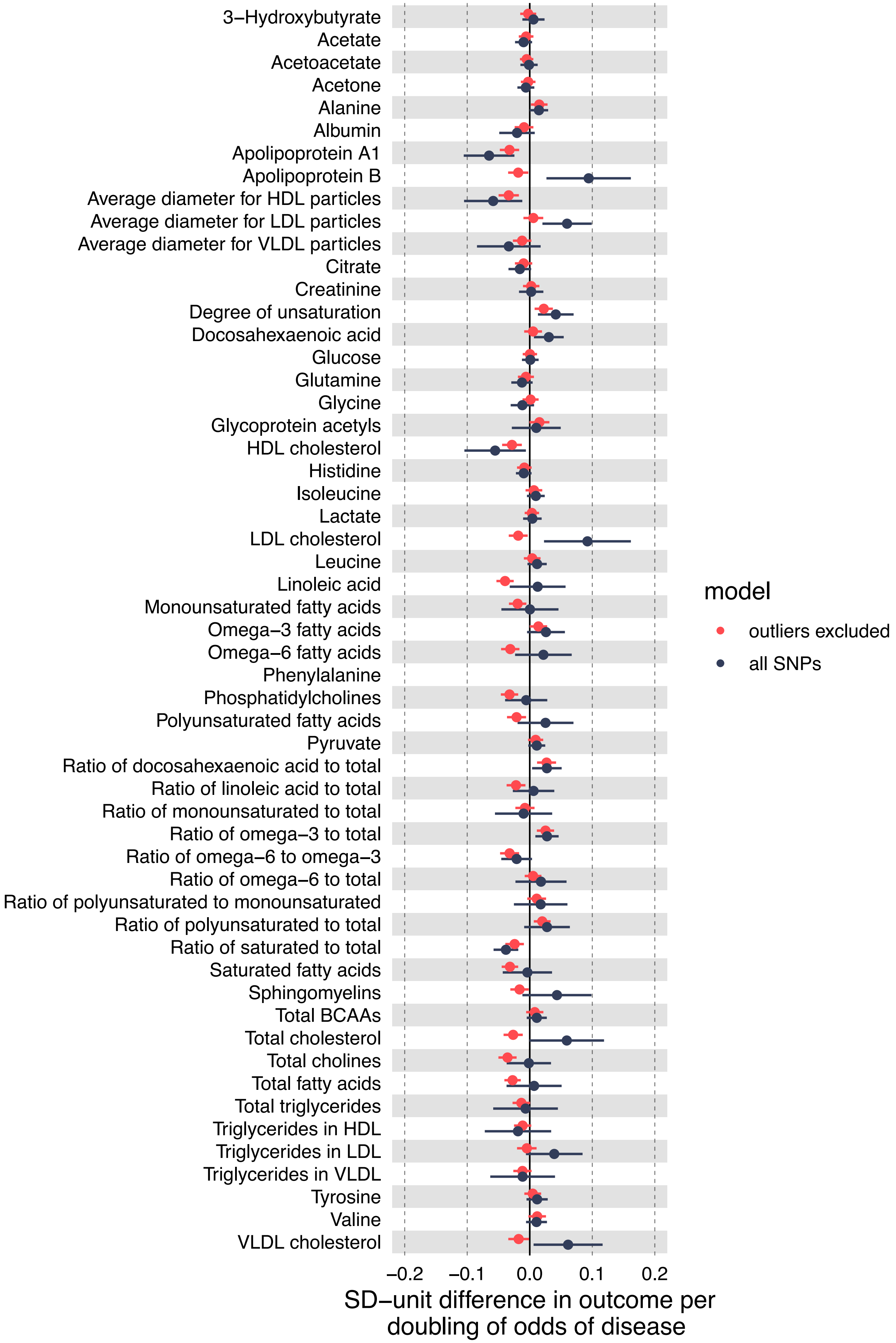

Supplementary Figure 11. Radial MR results of the effect of CAD on metabolites. Effect estimates are SD-unit differences in metabolite per doubling of liability to CAD based on IVW models, with all SNPs (blue) versus with outlying SNPs excluded (red). Metabolites are in alphabetical order. CAD, coronary artery disease; SD, standard deviation; IVW inverse variance weighted.

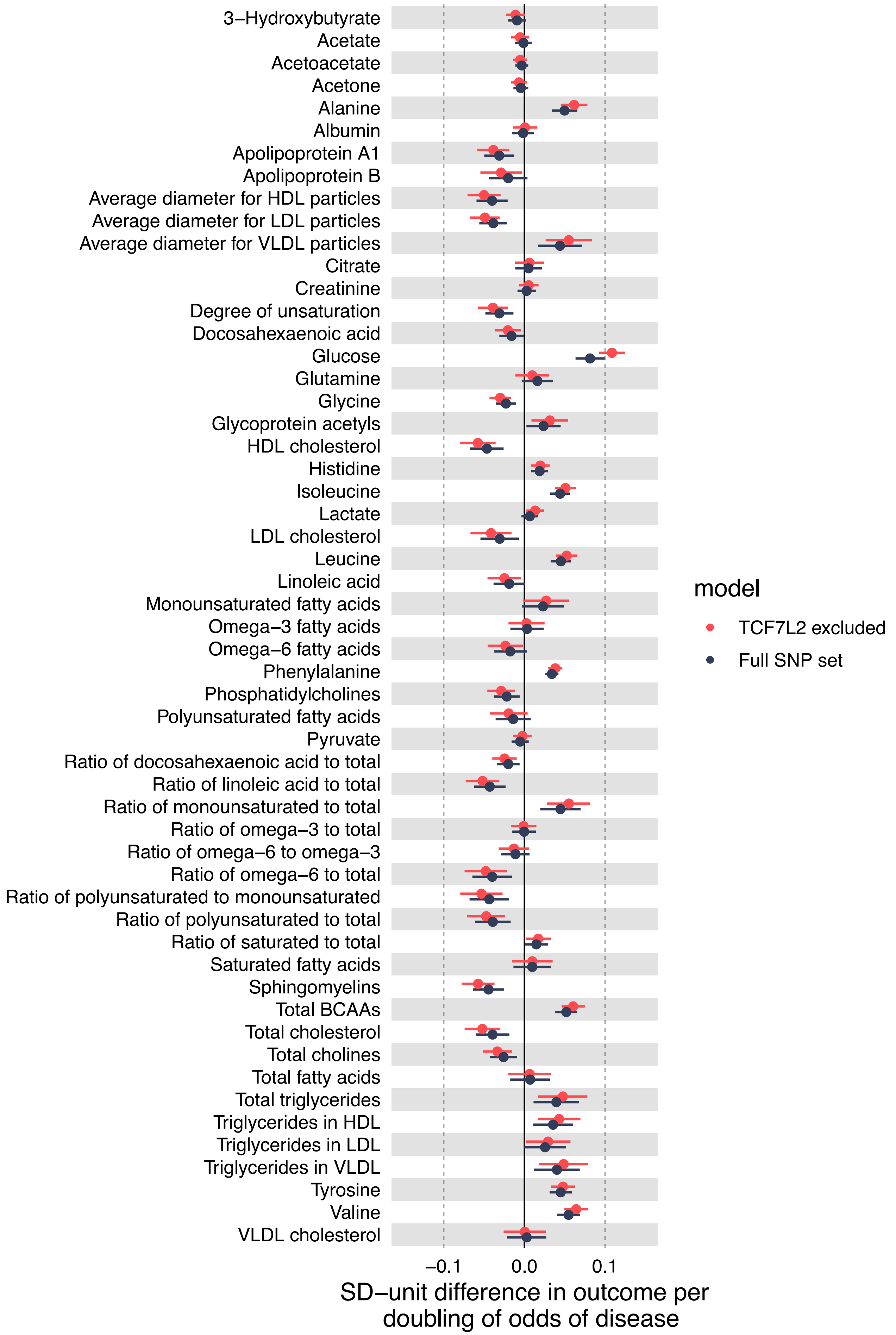

Supplementary Figure 12. Effect of T2D on metabolites. Effect estimates are SD-unit differences in metabolite per doubling of liability to T2D based on IVW models, with all SNPs (blue) versus with TCF7L2 variant excluded (red). Metabolites are in alphabetical order. T2D, type 2 diabetes; SD, standard deviation; IVW inverse variance weighted.

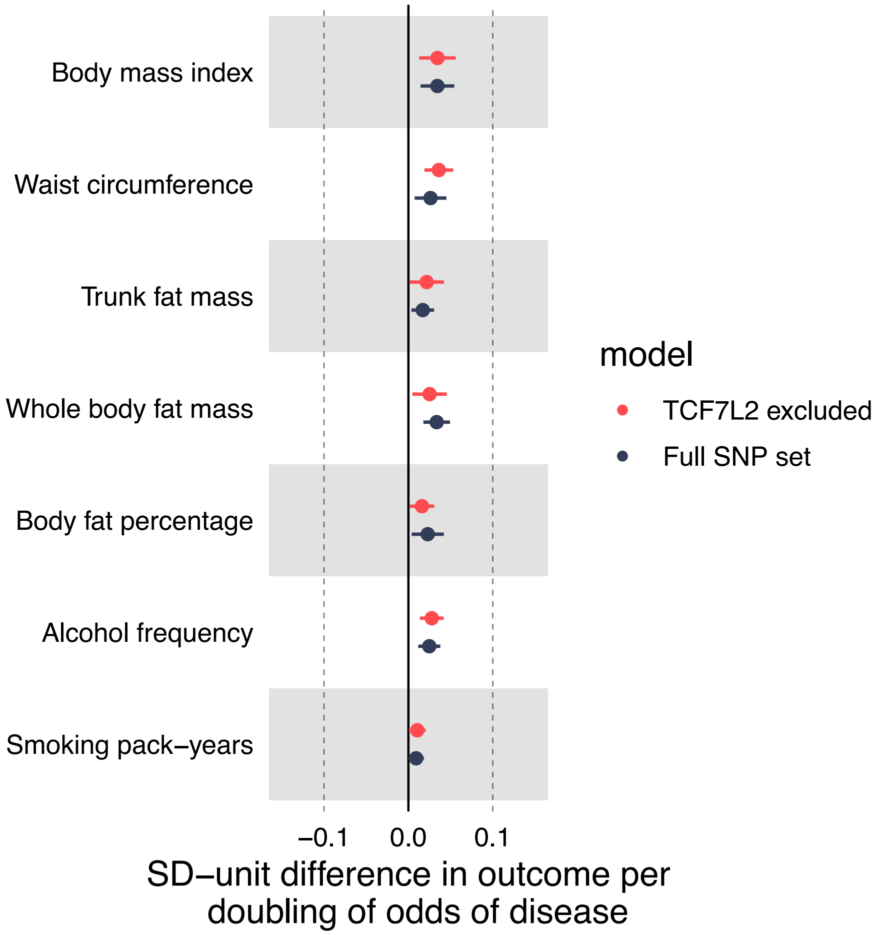

Supplementary Figure 13. Effect of T2D on adiposity and lifestyle outcomes. Effect estimates are SD-unit differences in outcome per doubling of liability to T2D based on IVW models, with all SNPs (blue) versus with TCF7L2 variant excluded (red). T2D, type 2 diabetes; SD, standard deviation; IVW inverse variance weighted.
